# Synthesis of Pyrimethanil-Loaded Mesoporous Silica Nanoparticles and Its Distribution and Dissipation in Cucumber Plants

**DOI:** 10.3390/molecules22050817

**Published:** 2017-05-16

**Authors:** Pengyue Zhao, Lidong Cao, Dukang Ma, Zhaolu Zhou, Qiliang Huang, Canping Pan

**Affiliations:** 1 Key Laboratory of Integrated Pest Management in Crops, Ministry of Agriculture, Institute of Plant Protection, Chinese Academy of Agricultural Sciences, No. 2 Yuanmingyuan West Road, Haidian District, Beijing 100193, China; pengyue_8825@163.com (P.Z.); caolidong@caas.cn (L.C.); 18800126646@163.com (D.M.); hbndzz191@163.com (Z.Z.); 2 Department of Applied Chemistry, College of Science, China Agricultural University, No. 2 Yuanmingyuan West Road, Haidian District, Beijing 100193, China

**Keywords:** mesoporous silica nanoparticles, pyrimethanil, distribution, dissipation, cucumber

## Abstract

Mesoporous silica nanoparticles are used as pesticide carries in plants, which has been considered as a novel method to reduce the indiscriminate use of conventional pesticides. In the present work, mesoporous silica nanoparticles with particle diameters of 200–300 nm were synthesized in order to obtain pyrimethanil-loaded nanoparticles. The microstructure of the nanoparticles was observed by scanning electron microscopy. The loading content of pyrimethanil-loaded nanoparticles was investigated. After treatment on cucumber leaves, the concentrations of pyrimethanil were determined in different parts of cucumber over a period of 48 days using high performance liquid chromatography tandem mass spectrometry. It was shown that the pyrimethanil-loaded mesoporous silica nanoparticles might be more conducive to acropetal, rather than basipetal, uptake, and the dosage had almost no effect on the distribution and dissipation rate in cucumber plants. The application of the pesticide-loaded nanoparticles in leaves had a low risk of pyrimethanil accumulating in the edible part of the plant.

## 1. Introduction

Cucumber (*Cucumis sativus* L.), one of the most popular fruiting vegetables, has a wealth of nutritional and health care value with a crisp taste [1]. Unlike the other vegetables, cucumbers, which are used as fresh vegetables or made into pickles, are typically harvested in the exponential phase of growth from the middle to the end, 7–14 days after pollination or about 14–21 days before maturation [2]. According to Food and Agriculture Organization, in 2014, cucumber production (including cucumbers and gherkins) was about 5.7, 1.8, 0.8 million tons in China, Russian, and USA, respectively [3]. However, the insects and diseases attacking cucumbers can reduce the vegetable quality, lower the yield, and shorten a life cycle, which causes serious loss of production to growers [4]. To ensure high-quality cucumber production, pesticides are applied by growers frequently during the production. As the cucumber is a kind of fresh-eating product, consumers may be exposed to the unsafe pesticide residues, thus posing a potential health risk. In 2016, to propose a ranking list for the popular fresh fruits and vegetables, the pesticide residue levels in 48 popular fruits and vegetables from the U.S. Department of Agriculture and Food and Drug Administration were investigated by the Environmental Working Group, an advocacy nonprofit. As a result, cucumber ranked 13 in all of the 48 items, which was listed from the worst to the best [5].

Due to various method of application and environmental conditions, only about 10% of pesticides are used on crops, as most of them are lost to the air or run off during application, not only polluting the environment but also raising production costs to growers [6]. Indiscriminate pesticide application on cucumber plants may increase pest resistance and pathogens, diminish nitrogen fixation, and reduce soil biodiversity, which leads to the destruction of habitats for non-target organisms and the bioaccumulation of pesticides, and causes potential health risks to human beings [7]. Therefore, it is important to reduce the excessive use of pesticides on cucumbers. Many studies have suggested that, in pesticide delivery applications of nanotechnology is a relatively new method and still in its infancy, which aims to reduce chemical pesticide abuse and guarantee their safety [8,9,10,11,12].

For many decades, nanotechnology has been applied in several areas, such as medicine, for both practical applications and research [13]. After Mobil Crystalline Material 41 was discovered, more and more researchers became interested in the research and application of mesoporous silica nanoparticles (MSNs). As it is possible to make dispersible, uniform, porous nanoparticles with colloidal chemistry and evaporation-induced self-assembly, MSNs were widely used to deliver proteins [14], genes [15,16], reactors [17], and drugs [18,19,20,21,22] as “nanocarriers”. However, in the case of the field of agriculture, only in recent years have researchers figured out the potential applications of nanotechnology. As it has many unique properties, such as a large surface area, biocompatibility, a tunable pore size for high loading capacity, a low cost, and an ability to control pesticide release, MSNs used as pesticide delivery could have a beneficial effect on environmental protection and decrease non-target insect exposure to pesticide [23,24,25]. 

In recent years, many studies have been reported about the uptake, absorption, and translocation of MSNs as pesticide carries by plants. For example, Hussain et al. reported the uptake by Arabidopsis, wheat, and lupin of MSNs functionalized with amine cross-linked fluorescein isothiocyanate and proposed that MSNs could be used to carry some small molecules in plants [26]; Chang et al. delivered foreign DNA into intact Arabidopsis thaliana roots using functionalized MSNs as carriers without the aid of mechanical force [15]; in our previous work, quaternized chitosan-capped MSNs were synthesized as nanocarriers for controlled pesticide release [27]. Nevertheless, in order to apply nanotechnology to the pesticide delivery systems, more systematic research results are important for the transport routes of pesticide-loaded MSNs. For instance, in which tissues or organs pesticide-loading MSNs tend to accumulate, how the distribution regulation of pesticide in plants will be after delivered by MSNs, and if pesticides tend to accumulate in the edible part of plant. Those kinds of studies are important in the case of using pesticide-loading MSNs in plants and could clarify the enrichment possibilities of the pesticides in grains or fruits and acquire a better understanding of the function of nanoparticle carriers on the translocation of pesticides in plants when they enter further into the food chain.

In the present work, pesticide-loaded MSNs were applied to plants to figure out its distribution and dissipation in cucumber plants. Pyrimethanil was chosen as the template pesticide, since it was widely used to control gray mold disease in cucumbers. MSNs with diameters of 200–300 nm were synthesized in order to obtain pyrimethanil-loaded MSNs (Py-MSNs). After treatment of pyrimethanil-loaded MSNs on cucumber leaves, the concentrations of pyrimethanil were determined in different parts of cucumber over a period of 48 days. Moreover, the final residue level of pyrimethanil in cucumber fruits was also explored to provide experimental evidence for the safety evaluation of pesticide-loaded MSNs in cucumbers.

## 2. Results and Discussions

### 2.1. Preparation and Characterization of MSNs and Py-MSNs

**Figure 1:**
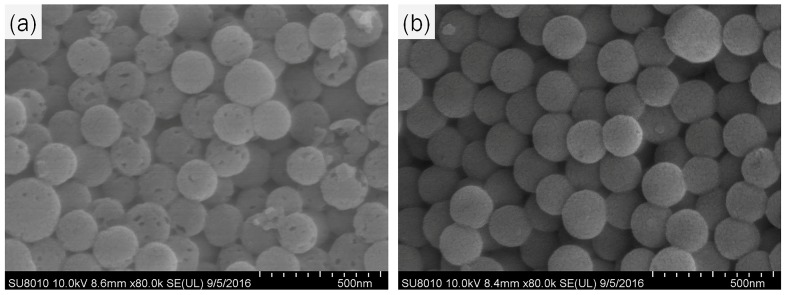
SEM images: (**a**) MSNs; (**b**) pyrimethanil-loaded MSNs.

**Figure 2:**
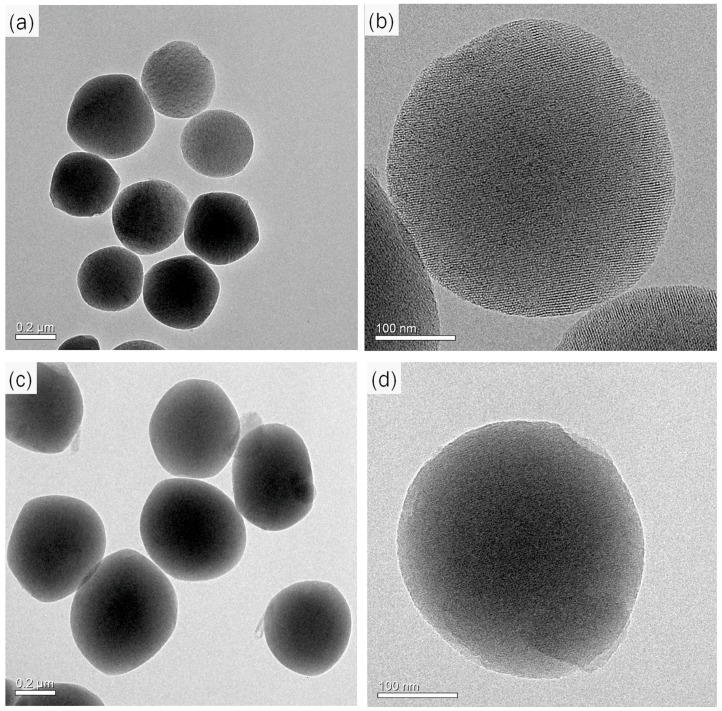
SEM images: (**a**,**b**) MSNs; (**c**,**d**) pyrimethanil-loaded MSNs.

In this study, the liquid crystal templating mechanism was used to prepare MSNs with TEOS as the silica precursor and CTAB as the surfactant in basic conditions. Pesticide molecules were loaded into MSNs by simple immersion in a concentrated toluene solution of pyrimethanil. The morphology of the MSNs and Py-MSNs was observed using SEM and TEM. As shown in Figure 1, SEM images showed that there was no obvious difference between the MSNs and Py-MSNs, since both of them had average diameters of 200–300 nm and a monodispersed spherical structure. In order to evaluate the mesoporous structure of nanoparticles before and after pesticide-loading, TEM was used to observe their physical characteristics confirming the MSN framework. In Figure 2a,c, it can be seen that MSNs and Py-MSNs have similar structures, average diameters, and dispersion, which verified the results in the SEM image. It can be seen in Figure 2b that there is a highly ordered mesoporous structure, one of the typical structures of MSNs, on the surface of the MSNs before pyrimethanil loading. However, for Py-MSNs, the mesoporous structures are not obvious in Figure 2d. This suggests that pyrimethanil was successfully loaded onto the MSNs without destroying the structure of the nanoparticles, and most of the mesoporous structures were blocked by pyrimethanil.

In practical applications, it is important to obtain relatively high loading concentrations of pesticide in controlled and sustained formulations during plant protection. The mesoporous structure, surface area, and pore size of MSNs would have a great effect on the release and uptake behaviors of the loaded compounds [28]. In our study, the solvent would change the pesticide loading. In order to obtain high loading concentrations, three solvents—chloroform, toluene, and hexane—were used to dissolve pyrimethanil for its loading, and the loading content levels were 3.92%, 29.77% and 20.56%, respectively. Therefore, toluene was used as the solvent for Py-MSN preparation.

**Figure 3:**
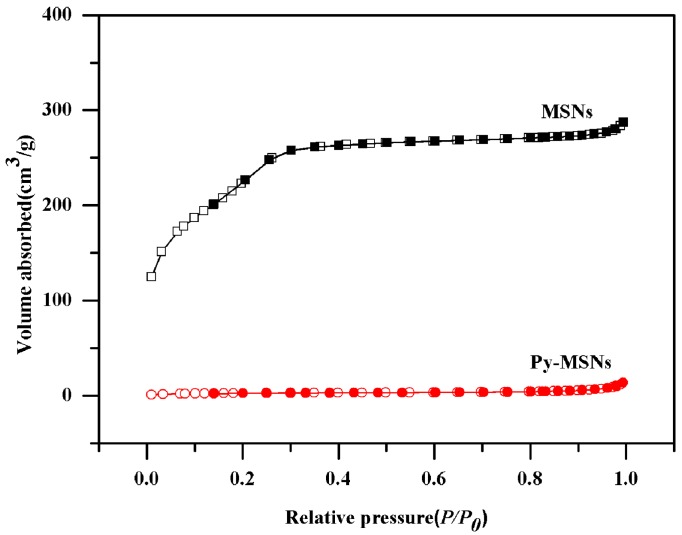
Nitrogen adsorption–desorption isotherms of MSNs and Py-MSNs.

In order to evaluate the pyrimethanil-loading potential, Brunauer–Emmett–Teller (BET) surface area analysis and Barrett–Joyner–Halenda (BJH) pore size and volume analysis were used to confirm the mesoporous structure of the nanoparticles. After pyrimethanil-loading on the MSNs, the BET specific surface area of the nanoparticles decreased sharply from 808.90 to 9.47 m^2^/g. As shown in Figure 3, the type IV isotherm curve of MSNs increased gradually from 0 to 0.3 of *P*/*P*_0_, and Py-MSNs showed an obvious reduction in adsorption capacity and surface area since the mesoporous was totally blocked by pyrimethanil.

### 2.2. In Vitro Release of Pymethanil

**Figure 4:**
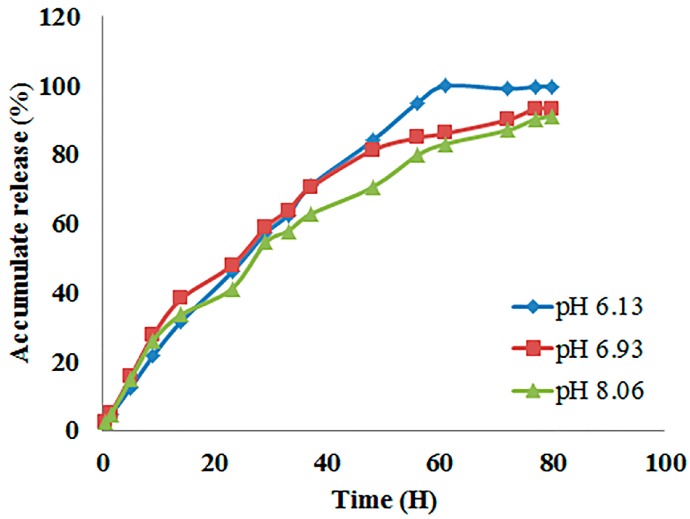
Release rates of pyrimethanil from Py-MSNs in pH 6.13, 6.93, and 8.06 at room temperature.

To simulate the environment of the pesticide delivery process, three different phosphate buffers (pH 6.13, 6.93, and 8.06) were used as the respective release medium for the cucumbers, roots, and leaves, respectively. As shown in Figure 4, from 0 to 30 h, the percentages of released pyrimethanil associated with the three pH values were similar and gradually increased to about 56%. Then, after the 30th hour, when the pH value was 6.13, more pyrimethanil was released than those with pH values of 6.93 and 8.06. Finally, 99% of pyrimethanil was released after 60 h when the pH of the release medium was 6.13, and it then kept a release balance. In the case with pH values of 6.93 and 8.06, the pesticide-loading nanoparticles released 93% and 91% of pyrimethanil after 80 h, respectively. This indicated that pyrimethanil in the nanoparticles would release faster than that in cucumber leaves and roots, and it would release the slowest in leaves.

### 2.3. Analytical Method Validation

As EU guidelines recommend [29], to exclude any results produced by matrix effects, matrix-matched calibration solutions were used to test the linearity of the method for more accurate results. Matrix-matched calibration solutions was obtained from blank extracts with pyrimethanil in the range of 2–200, 2–200, and 0.2–20 μg/L for leaves, root, and cucumber respectively. The quantitative results of the analysis method were mainly dependent on linearity. As shown in Table 1, a good linearity was obtained for pyrimethanil with coefficients of determination (R^2^) better than 0.995.

In this study, recovery was the amount measured as a percentage of the amount of pyrimethanil originally spiked to the blank root or leaves samples. The repeatability of the proposed method was expressed as a relative standard deviation (RSD, %, n = 5). The precision and accuracy data of the proposed method was investigated for pyrimethanil spiked at three fortified levels of 0.01, 0.1, and 1 mg/kg in the blank leaf and root samples; 0.001, 0.01, and 0.1 mg/kg in blank cucumber samples. For each concentration, five spiked tests were repeated for each matrix (n = 5). Limits of quantification (LOQs), average recoveries, and relative standard deviations are shown in Table 1. The recoveries of pyrimethanil were in the range 78–85% for cucumber leaves, 87–99% for roots, 74–90% for cucumbers, and RSDs were no more than 10% for all cases. LOQs were determined as the concentration of pyrimethanil, giving a signal to noise ratio (S/N) of 10 for the target ion, which was calculated with Agilent Mass Hunter Qualitative Analysis software.

### 2.4. Distribution of Pyrimethanil-Loaded Mesoporous Silica Nanoparticles in Cucumber Plant

The results of the study revealed that treatment of Py-MSNs on cucumber leaves resulted in acropetal and basipetal uptake, and the distribution and translocation of the chemical to different cucumber plant parts, i.e., the roots and the upper, lower, and treated leaves. It could be seen in Figure 2a,b that, three days after treatment of Py-MSNs on cucumber plants, the concentration of pyrimethanil increased to 0.210 and 0.422 mg/kg in the upper leaves, and then declined gradually to 0.011 and 0.013 mg/kg when 0.5 and 2 mg/mL of Py-MSNs were used, respectively. Similarly, in the lower leaves and roots, the concentration of pyrimethanil increased first and then descended, which is shown in Figure 2c–f. In general, it was higher in the upper leaves than in the lower leaves and roots. As long as seven days after treatment, the highest concentration of pyrimethanil was reached in the roots, and compared to upper and lower leaves, the concentrations of pyrimethanil in the roots were the lowest. This shows that Py-MSNs might be more conducive to acropetal, rather than basipetal, uptake in cucumber plants.

**Figure 5:**
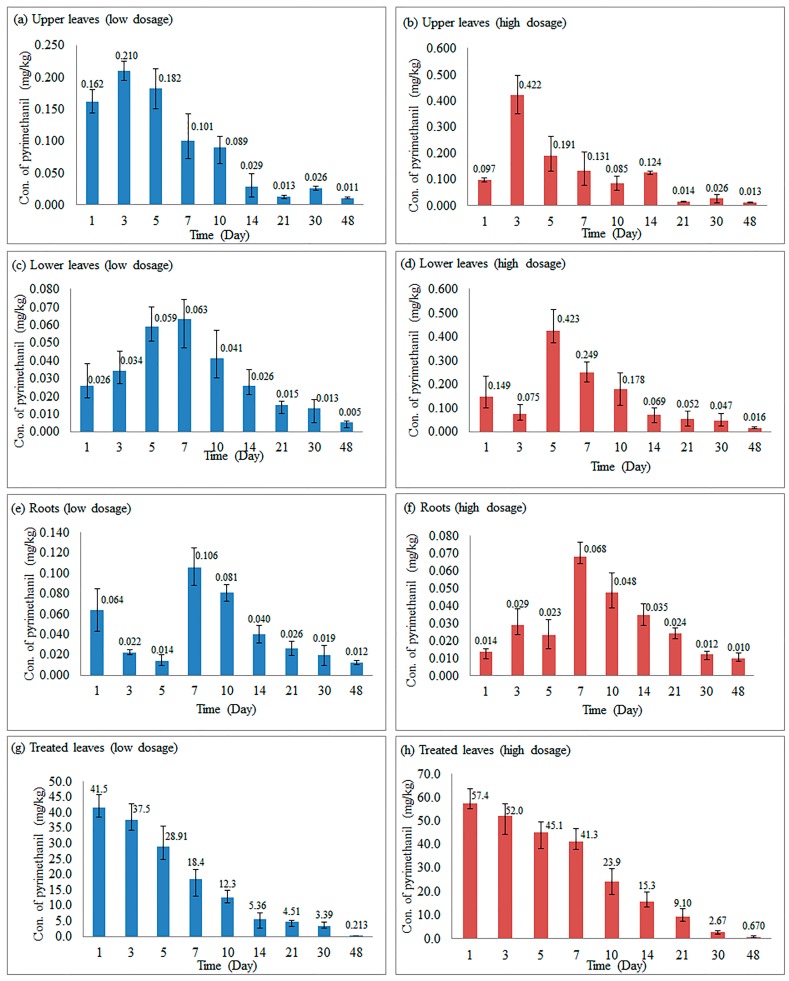
Distribution of Py-MSNs in cucumber plant: (**a**) upper leaves with 0.5 mg/mL treatment; (**b**) upper leaves with 2 mg/mL treatment; (**c**) lower leaves with 0.5 mg/mL treatment; (**d**) lower leaves with 2 mg/mL treatment; (**e**) roots with 0.5 mg/mL treatment; (**f**) roots with 2 mg/mL treatment; (**g**) treated leaves with 0.5 mg/mL treatment; (**h**) treated leaves with 2 mg/mL treatment.

In this study, two dosages of 0.5 and 2 mg/mL of Py-MSNs were chosen to explain the distribution of pyrimethanil in cucumber plant. As can be seen in Figure 5, generally, the concentrations of the chemical were higher when 2 mg/mL of Py-MSNs was used compared to 0.5 mg/mL of Py-MSNs. However, there were no obvious differences in the distribution of pesticides between 0.5 and 2 mg/mL, which showed that the dosage had no effect on the distribution of Py-MSNs in cucumber plants.

### 2.5. Dissipation of Pyrimethanil-Loaded Mesoporous Silica Nanoparticles in Different Parts of Cucumber Plants

The half-lives and decline regression equations of pyrimethanil from Py-MSNs are summarized in Table 2. The dissipation curves of pyrimethanil were carried out in upper, lower, and treated leaves and roots for 3–48, 5–48, 1–48, and 7–48 days, respectively, which followed the first-order kinetic reaction. As shown in Table 2, the decline of pyrimethanil in the treated leaves was drastic as compared to that in the upper or lower leaves or the roots, as the half-lives were the shortest as 6.5 and 7.0 days at low and high dosage levels, respectively. Most Py-MSNs were dropped onto the treated leaves, and their pyrimethanil concentrations were far higher than those of the other leaves, as shown in Figure 2g,h. The cucumber plant could absorb and transport only a limited amount of the pesticide solution and left most of pyrimethanil on the surface of the treated leaves. The pyrimethanil that are not absorbed by cucumbers would degrade rapidly in the environment as time went on. In the case of roots, the half-life of pyrimethanil was 20.6 and 21.7 days for the low and high dosages, respectively, which is longer than that of the upper, lower or treated leaves. This shows that Py-MSNs had the slowest dissipation rate in cucumber roots instead of the leaves. Moreover, the half-life in the four parts of the cucumber plant did not show any obvious differences between the low and high dosages of treatment. This illustrates that the dissipation rate of Py-MSNs might not change by variation of the treatment dosage.

Pyrimethanil has been widely used to control gray mold disease on cucumbers all over the world. Many studies about the dissipation of pyrimethanil in classical formulations on plants have been carried out. For example, Malhat et al. found that the half-life of wettable pyrimethanil powder on cucumbers was 2.19 days [30], and Liu et al. showed that the half-lives of emulsifiable pyrimethanil concentrates on tomato were 1.8–4.2 days [31]. Compared to these references, the dissipation of pyrimethanil was much slower when Py-MSNs was used. According to the release test in vitro, 91% of pyrimethanil was released after 80 h on leaves. As pyrimethanil was held in MSNs, the mesoporous structure controlled its release to the environment.

### 2.6. Final Residues of Pyrimethanil in Cucumber

The final residue results showed that, in the fruiting stage, the concentrations of pyrimethanil in cucumbers were 0.003, 0.002, and 0.002 mg/kg with a low dosage level of 0.5 mg/mL, and 0.004, 0.005, and 0.003 mg/kg with the high dosage level of 2 mg/mL. The maximum residue limit (MRL) established by Codex, United States, Europe Union, China, and Japan for pyrimethanil in cucumber is 0.7, 1.5, 0.7, 2, and 2 mg/kg, respectively. It was obvious that the concentration levels of pyrimethanil in cucumber fruit was much lower than the international MRL value after the leaves were treated by pyrimethanil-loaded MSNs. It is predicted that the application of Py-MSNs in leaves has a low risk of pyrimethanil accumulating in the edible part of the plant.

## 3. Materials and Methods

### 3.1. Materials

Cetyltrimethylammonium bromide (CTAB, 99%) and tetraethyl orthosilicate (TEOS) were purchased from J&K Scientific Ltd., Beijing, China, and Fluorochem Ltd., Hadfield, UK, respectively. The template pesticide, pyrimethanil (98.0%), was supplied by Jiangsu Weunite Fine Chemical Co., Ltd. Acetonitrile of HPLC grade was purchased from Fisher Scientific (Geel, Belguim). Deionized water was obtained from a Milli-Q water purification system from Millipore, USA. Graphitized carbon black (GCB) and primary secondary amine (PSA) were purchased from Agela Technologies, Tianjin (China).

### 3.2. Synthesis of Mesoporous Silica Nanoparticles

Mesoporous silicas nanoparticles were first successfully synthesized and published by the groups of Cai [32], Mann [33], and Ostafin [34]. After that, various synthesis methods were published to prepare nanoparticles in good dispersion and mesoporous silica nanoparticles with various structures and tunable dimensions from a handful to hundreds of nanometers [35,36]. In order to synthesize MSNs, in basic conditions, TEOS and CTAB were used as the silica source and structure-directing agent, respectively, referring to Radu [37]. Four hundred eighty milliliters of deionized water was used to dissolve 1.0 g of CTAB. Then, under constant stirring, 3.5 mL of sodium hydroxide (2.0 M) was added into the CTAB solution at room temperature. After the mixture was heated to 70 °C in an oil bath, 5.0 mL of TEOS was introduced drop by drop at the rate of 1.0 mL/min, and it was then stirred vigorously at 80 °C for 6 h. During the procedure, MSNs were formed as a white solid, which was washed by ethanol and deionized in water three times and freeze-dried under vacuum. In order to remove the surfactant completely, the synthesized MSNs was then calcined at 550 °C for 6 h. Electron microscopic studies were carried out by a scanning electron microscopy (SEM) (SU8000, Hitachi, Ltd., Tokyo, Japan) and transmission electron microscopy (TEM) (JEM-200CX, Jeol Ltd., Tokyo, Japan) to study the internal structure of Py-MSNs and MSNs.

### 3.3. Loading of Pyrimethanil into Mesoporous Silica Nanoparticles

Thirty milligrams of synthesized MSNs was added into the pyrimethanil-toluene solution (6.0 mg/mL, 5.0 mL). The mixture was magnetic stirred for 6 h, and the supernatant was then removed by centrifugation at 10,000 rpm for 10 min. The pyrimethanil-loaded MSNs was dried at 50 °C for 5 h to remove the supernatant completely.

The pyrimethanil loading efficiency of the proposed method was determined by high performance liquid chromatography (HPLC, 1200-DAD (Diode Array Detector), Agilent, Santa Clara, CA, USA). In brief, 10.0 mg of Py-MSNs were dissolved in 25.0 mL of methanol under sonication for 2 h. This process was repeated several times, and the methanol solution was combined for HPLC analysis, until the concentration level of pyrimethanil in the solution lower than the limit of detection.

For the HPLC analysis, Venusil XBP-C18 column (Bonna-Agela Technologies Inc., Tianjin, China) (2.5 mm × 4.6 mm, 5 µm) was used to separate the target compound from others at 30 °C. Methanol/water (80/20) was used as mobile phase at a flow rate of 1.0 mL/min. DAD signals was used as 275 nm. Loading efficiency (%) = (weight of pesticide in nanoparticles/weight of nanoparticles) × 100%. In this study, the loading efficiency of MSNs was 29.77%.

The nitrogen adsorption of MSNs and Py-MSNs was studied to show the typical pore characteristics using a surface area and pore size analyzer (TriStarII 3020, Micromeritics Instruments Corp, Norcross, GA, USA) at 196 °C. Samples were degassed at 80 °C for 12 h prior to analysis. The characteristics of mesoporous structure were analyzed by the BET and BJH procedures from the adsorption branches of the isotherms.

### 3.4. Pyrimethanil Release

Twenty milligrams of Py-MSNs were dispersed in 250 mL of phosphate buffer (pH 6.13, 6.93, 8.06) with 0.1% Tween-80 emulsifier, which was used as the release medium. A D-800LS dissolution tester (Tianjin University, Tianjin, China) was used for the release test at a stirring speed of 120 rpm. In order to verify the sustained release property of Py-MSNs, the cumulative release rate of pyrimethanil from the nanoparticles was calculated with its concentration in the release medium. For HPLC analysis, 0.8 mL of the release medium was withdrawn at given time intervals, and the same volume of fresh buffer solution was supplied to ensure the volume of release medium. The determination was repeated twice. The accumulative release was calculated as followed:

(1) $$E_{r}=\frac{V_{e}\sum_{1}^{n-1}C_{i}+V_{0}C_{n}}{M_{p}}\times 100\%$$

*E_r_*: the accumulative release (%) of pyrimethanil from the nanoparticles;
*V_e_*: the volume of the release medium taken in a time interval (*V*_e_ = 0.8 mL);
*C_i_*: the pyrimethanil concentration in release medium;
*i*: release time
*V*_0_: the volume of release medium (250 mL);
*n*: the sample number;
*M_p_*: the total amount of pesticide enwrapped in the particles.

### 3.5. Greenhouse Study

**Figure 6:**
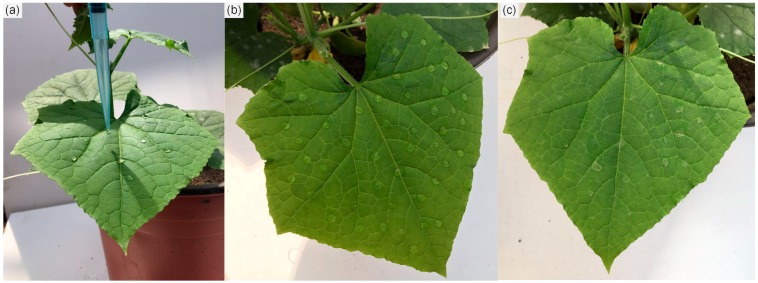
Treatment of Py-MSNs on leaves (**a**) Py-MSNs treatment using a pipette; (**b**) 2 h after treatment.

**Figure 7:**
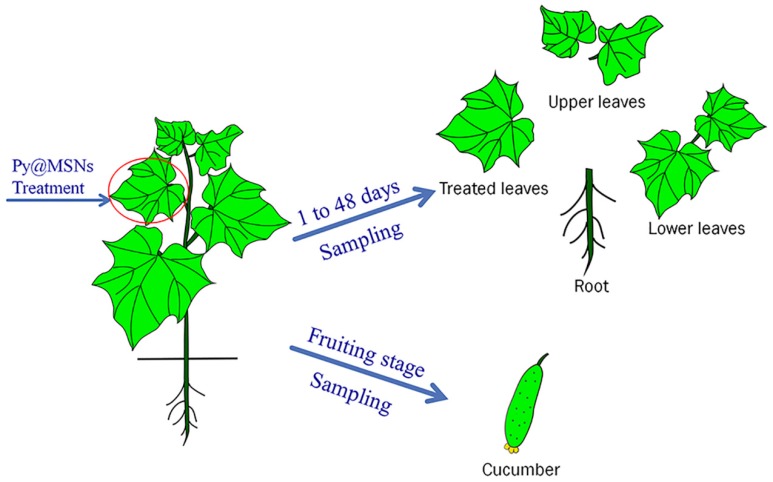
Sampling for the study of pyrimethanil distribution in cucumbers.

For the study, non-stained cucumber seedlings were used, which were produced by the Institution of Plant Protection, Chinese Academy of Agricultural Sciences. Cucumber plants were cultivated in plastic pots with potting soil. The Py-MSNs were dispersed in sterile distilled water prior to exposure. Two concentration levels of pyrimethanil were used for the distribution study: 0.5 and 2 mg mL^−1^. When the cucumber seedlings had five to six leaves, 0.5 mL of Py-MSNs with two concentrations aqueous solution was added on one of the middle leaves from cucumber plant drop by drop using a pipette to stop the droplets from running off, shown in Figure 6. In order to figure out the residue behavior of pyrimethanil in cucumber plants during different growth durations, representative samples were collected 1, 3, 5, 7, 10, 14, 21, 28, 30, and 48 days after Py-MSNs treatment on cucumber plants. Each treatment was repeated three times. As shown in Figure 7, the concentrations of pyrimethanil were measured in four parts of cucumber plant, i.e., the roots and leaves (upper, middle and lower), over a period of 48 days using high performance liquid chromatography tandem mass spectrometry (HPLC-MS/MS). Moreover, the concentration levels of pyrimethanil in cucumber fruits were also determined during the fruiting stage.

### 3.6. Sample Preparation

In recent years, the QuEChERS (quick, easy, cheap, effective, rugged, and safe) method was published by Anastassiades et al. in 2003 [38,39], which is now widely used as pesticide analysis methods in fruit and vegetables by many governments and organization laboratories. For sample preparation, a modified QuEChERS method was employed to analysis the concentration of pyrimethanil in cucumbers. In brief, 2.0 g of homogenized samples were extracted with 10.0 mL of acetonitrile, and 4.0 g of anhydrous magnesium sulfate and 1.0 g of sodium chloride was then introduced for salting out. After that, the loose sorbents of 5 mg of GCB, 25 mg of PSA, and 150 mg of MgSO_4_ was used to clean up 1.0 mL of acetonitrile extract. For treated leaves, the acetonitrile extraction was diluted 20 times before HPLC-MS/MS detection.

The concentration levels of pyrimethanil in different parts of the cucumber plants and fruits were determined by an Agilent 1200 HPLC equipped with a reversed-phase column (ZORBAX SB-C18, 3.5 μm, 2.1 mm × 50 mm, Agilent, Santa Clara, CA, USA) at 25 °C. Acetonitrile/water (80/20, *v*/*v*) was used as a mobile phase at a flow rate of 0.3 mL/min^−1^. The injection volume was 5 μL. An Agilent 6410 Triple Quad LC/MS system was applied for mass spectrometric analysis. Electrospray ionization source in positive ionization mode was used for pyrimethanil analysis. Nitrogen gas was used as both the collision and nebulizer gas. The other parameters of mass spectrometry were as follows: gas flow: 8 L min^−1^; gas temperature: 350 °C; capillary voltage: 4000 V; and nebulizer gas: 35 psi. The multiple reaction monitoring mode was used to monitor the precursor to product ion transitions. The retention time of pyrimethanil was 0.9 min. Two ion transitions were chosen: *m*/*z* 200→*m*/*z* 107 (quantification), and *m*/*z* 200→*m*/*z* 183 (confirmation). Agilent Mass Hunter Data Acquisition and Qualitative Analysis and Quantitative Analysis software was used for method development and data acquisition.

## 4. Conclusions

Nowadays mesoporous silica nanoparticles are used as pesticide carries in plants and have been considered as a novel method to reduce the indiscriminate use of conventional pesticides. However, more and more misgivings are the translocation, distribution, and dissipation of those nanoparticles in plants, which limits their wide-scale applications. In this study, pyrimethanil-loading MSNs were synthesized and applied to cucumber leaves. It was shown that Py-MSNs might be more conducive to acropetal, rather than basipetal, uptake in cucumber plants, and the dosage had almost no effect on distribution and dissipation rate of Py-MSNs in cucumber plants. The application of Py-MSNs in leaves would have a low risk of pyrimethanil accumulating in the edible part of the plant. The work of this study supplied new information on the further understanding of the distribution and dissipation of pesticide-loaded MSNs applied as foliar treatments. This information adds to our knowledge of the uptake and translocation of pesticides in plants and helps to explain the effects of nanoparticle carriers on the translocation of pesticides in plants. This research provides insight into the possibilities of pesticide-loaded MSNs accumulating in fruits or grains for further entry into the food chain.

## Figures and Tables

**Table 1:** Average recoveries, RSDs (n = 5), LOQs, linear equation, and determination coefficients (R^2^) of pyrimethanil in leaves, roots, and cucumber.

Sample	Fortified Level (mg/kg)	Average Recoveries (%)	RSD (%)	LOQ (mg/kg)	Linear Equation	R^2^
Leaf	0.01	78	8	0.004	*y* = 3,964,048*x* + 9802	0.993
	0.1	82	9			
	1	85	6			
Root	0.01	87	5	0.001	*y* = 5,007,122*x* + 3234	0.998
	0.1	94	7			
	1	99	8			
Cucumber	0.001	74	10	0.001	*y* = 4,817,122*x* + 1329	0.996
	0.01	90	7			
	0.1	87	8			

**Table 2:** The half-life and other statistical parameters for the dissipation of pyrimethanil-loaded MSNs in cucumber plants.

Part	Day	Dosage (mg/mL)	Regression Equation	Determination Coefficient (R^2^)	Half-life (Days)
Upper leaves	3–48	0.5	*y* = 0.1243e^−0.064(*x* − 3)^	0.7277	13.8
		2	*y* = 0.1934e^−0.073(*x* − 3)^	0.7484	12.5
Lower leaves	5–48	0.5	*y* = 0.0540e^−0.059(*x* − 5)^	0.9484	16.7
		2	*y* = 0.2417e^−0.068(*x* − 5)^	0.8671	15.2
Root	7–48	0.5	*y* = 0.0757e^−0.051(*x* − 7)^	0.8703	20.6
		2	*y* = 0.052e^−0.047(*x* − 7)^	0.8893	21.7
Treated leaves	1–48	0.5	*y* = 42.407e^−0.106*x*^	0.9582	6.5
		2	*y* = 68.458e^−0.099*x*^	0.9916	7.0

## Abbreviations

MSNs	mesoporous silica nanoparticles
Py-MSNs	pyrimethanil-loaded MSNs
CTAB	cetyltrimethylammonium bromide
TEOS	tetraethyl orthosilicate
PSA	primary secondary amine
GCB	graphitized carbon black
SEM	scanning electron microscopy
DAD	diode array detector
HPLC-MS/MS	high performance liquid chromatography tandem mass spectrometry
QuEChERS	quick, easy, cheap, effective, rugged and safe
RSDs	relative standard deviations
LOQs	limits of quantification
MRL	maximum residue limit
